# Supplementary material for: A Narrative Review on Manufacturing Methods Employed in the Production of Mesenchymal Stromal Cells for Knee Osteoarthritis Therapy
Source: Biomedicines. 2025 Feb 18;13(2):509. doi: 10.3390/biomedicines13020509 (PMC11853043; doi:10.3390/biomedicines13020509)
Supplement: Supplementary file 1 [file biomedicines-13-00509-s001.zip › biomedicines-3395909-supplementary.pdf]

biomedicines

MDPI

Supplementary Table S1: Overview of outcome measures, adverse events and clinical efficacy in the clinical trials

No.	Author	Study design	Treatment / control (n)	KL grade	Intervention	Control	Outcome measures	Adverse events	Clinical efficacy
1	Kim et al [1]	RCT	131 / 130	III	sIAI of AD-MSC	sIAI of 2.1 mL of normal saline and 0.9 mL auto serum	VAS, WOMAC, KOOS, SF-36, IKDC, X-ray, MRI (WORMS)	Incidence of AEs: 38.4% in the intervention group and 32.3% in the control group. Unrelated SAE occurred in 1 patient in the intervention group (pneumonia) and 3 in the control group (COVID-19, Herpes zoster, and spondylolisthesis).	**VAS, WOMAC, KOOS, SF-36, IKDC scores:** Greater improvement from baseline in the intervention group than in the control group ($p<0.05$). **X-ray, MRI:** No significant change from baseline between the groups.
2	Günay et al [2]	CS	10	NS	sIAI of UC-MSC	NA	VAS, WOMAC, Lequesne Index, SF-36, qMRI, MRI (MOAKS), synovial levels of TNF-α, IL-6, IL-1β, adiponectin, resistin, and leptin	Incidence of AE: 33% (all knee effusion).	**VAS, WOMAC, SF-36 scores:** Significant improvement after 12 months follow-up. **Synovial leptin** levels were significantly decreased after 6 months follow-up.
3	Samara et al [3]	Prospective open-label clinical trial	16	III-IV	2xIAI of UC-MSC, interval of 1 month	NA	KOOS, MRI (semi-quantitative assessment of cartilage, bone marrow lesions, osteophytes, subchondral sclerosis, joint effusion and synovitis)	Incidence of AEs: 87%. The most common adverse event was knee pain. There were no SAE.	**KOOS score:** Compared to baseline the KOOS score was significantly improved at 6- and 12-months follow-up. This improvement was maintained after 48 months. **MRI:** Compared to baseline the MRI parameters (cartilage, bone marrow lesions, osteophytes, subchondral sclerosis, joint effusion and synovitis) were significantly improved at 6- and 12-months follow-up.
4	Kim et al [4]	CS	11	II-IV	sIAI of AD-MSC	NA	VAS, WOMAC, intervention performed in the follow-up period to the index knee, X-ray, MRI (cartilage defect, WORMS)	Incidence of AEs: 100% of which the majority were mild-moderate. Most common adverse events were arthralgia, back pain and hypertension. One SAE occurred in the follow-up period but was unrelated to the treatment as the patient had undergone spine surgery.	**VAS, WOMAC scores:** Scores were significantly improved after 12, 24, 36, 48 and 60 months. For the WOMAC Stiffness score, a significant improvement was noted at 12, 24 and 36 months but not after 48 and 60 months. **Interventions in the index knee:** During the 5-year follow-up, 9 patients had additional intra-articular injection of HA more than 1 year after MSC injection or administration of NSAIDs because of knee discomfort. During the 5-year follow-up period, none of the patients underwent any surgical intervention of the affected knee. **X-ray:** There were no changes in x-ray features after 6 months. **MRI (cartilage defect):** Compared to baseline the area of cartilage defect in the medial femoral condyle tended to decrease until 3 years after the injection and was

									maintained without significant aggravation until 5 years follow-up. **MRI (WORMS):** Compared to baseline the WORMS score significantly improved until 2-3 years.
5	Jianrui et al [5]	Retrospective study	40 / 46	0-II	3xIAI of BM-MSC, interval of 1 month	AD followed by 5xIAI of 2 mL HA, interval of one week between injections	VAS, WOMAC total effective rate, Serum TNF-α and IL-6	NS	**VAS score:** Significantly reduced from baseline after 6 and 12 months in both groups. When comparing the intervention and control group the VAS score was significantly lower in the intervention group at both 6 and 12 months. **WOMAC score:** Used to estimate the total effective rate between groups. The intervention group had a significantly higher total effective rate of 70% vs. 45.65% in the control group. **TNF-a and IL-6:** Compared to baseline, levels of both cytokines were significantly lower after 6 and 12 months in both groups and were significantly lower at 12 months compared to 6 months. Comparing the intervention group with the control group, the levels of both cytokines were significantly lower in the intervention group at both 6 and 12 months.
6	Lu et al [6]	Double-blinded, dose-ranging RCT	(1) low dose group n=7, (2) mid dose group n=8, (3) high dose group n=7	II, III	2xIAI of AD-MSC, for all dose groups, interval of 3 weeks	NA	VAS, WOMAC, SF-36, Laboratory assessment: CBC, urine routine, liver function, renal function, blood lipids, blood sugar, immunology, 12-lead ECG and vitals, MRI (WORMS), qMRI	Incidence of AE: 71.42%, 87.50%, and 100% in the low-dose group, mid-dose group, and high-dose group, respectively. Most common AEs were mild to moderate pain and swelling at the injection site (resolved within 3 days). No changes on ECG, vital signs, or physical examination. No abnormal biochemical results (baseline, week 1,3,4,8,12). Reasons for discontinuation: knee pain (n=2), new diagnosis of gallbladder carcinoma (n=1). 2 serious AEs reported, and both assessed unrelated to the investigational product (one with gallbladder carcinoma, one with thyroid carcinoma).	**VAS score and WOMAC:** Improvement was noted for all dose groups, however only significantly in the low dose and mid dose groups. **MRI:** across all dose groups there were mixed results, with some improvement in average cartilage volume noted in the low dose group.
7	Dilogo et al [7]	Prospective open-label clinical trial	29	I-IV	3xIAI, 1 injection UC-MSC, 2nd and 3rd injection 2 mL HA, interval of 1 week	NA	VAS, WOMAC, IKDC, qMRI	NS	**VAS score:** Reduced for all patients over the 12 months follow-up period but a significant reduction was only observed after 6 months in patients with KL III-IV. **WOMAC, IKCD score:** Compared to baseline, the WOMAC and IKDC scores improved significantly after 6 months in all patients. **qMRI:** Compared to baseline, the cartilage did not change after 6 and 12 months although a trend towards improvement was observed.

8	Chahal et al [8]	Prospective, open-label, dose-escalating clinical trial	(1) low dose group n=3, (2) mid dose group n=3, (3) high dose group n=9, mixed dose group (one patient low dose, one mid dose and one high dose)	III-IV	sIAI of BM-MSC for all groups	NA	KOOS, WOMAC, MRI (WORMS), MRI (synovitis), qMRI, Biomarkers: Blood (hyaluronan, cartilage oligomeric matrix protein, collagen type I and II), Urine (C-telopeptide of type II, collagen type II), Synovial fluid (Matrix metalloproteinases, tissue inhibitor of metalloproteinases, CX3CL1, CXCL1, CC2, VEGF, IL12p40, IL6, IL8, HGF, sCD163, sCD14, prostaglandin, adiponectin, adipsin, leptin, resistin)	Incidence of AEs: 100% and 33% in the high-dose group and the mixed-dose group (patient treated with the high dose). Most common adverse events were mild to moderate pain and swelling at the injection site (resolved within 2-4 weeks without treatment). There were no SAEs.	**KOOS and WOMAC scores:** Significantly improved after 12 months in nearly all subscale scores and in all groups but with the patients treated with the high dose having the greatest improvement. **MRI (WORMS), qMRI:** No statistically significantly change after 12 months follow-up in any of the groups. **MRI (synovitis):** Compared to baseline no significantly changes after 12 months follow-up. Between groups analysis showed that the high-dose group had a significantly lower level of synovitis than the low-dose group. **Biomarkers Blood and Urine:** Compared to baseline the biomarkers did not significantly change after 12 months. A trend towards lower increase in catabolic cartilage markers was noted in the high-dose group compared to the low-dose group. **Biomarkers; Synovial fluid:** Compared to baseline most of the factors remained unchanged after 3 months follow-up. A significant increase from baseline was observed in the VEGF level concomitant with a decrease in IL12p40 and adiponectin after 3 months. These changes were found across all doses.
9	Lu et al [9]	RCT	26 / 27	I-III	2xIAI of AD-MSC and 2 sham injections	4xIAI of 2.5 mL HA	VAS, WOMAC, SF-36, qMRI	Incidence of AEs: 53.85% in the control group and 73.07% in the intervention group. Most common AEs were mild to moderate pain and swelling at the injection site (resolved within 7 days). No changes on ECG, vital signs, physical examination and laboratory tests during the 12 months of follow-up. One SAE occurred in the control group with infection of knee joint after 2 months of first injection and the patient withdrew from the study. The SAE was relieved after articular cavity flushing operation.	**VAS score:** Compared to baseline significantly reduced after 6 and 12 months only in the intervention group. When comparing the intervention and control group the VAS score was significantly lower in the intervention group at both 6 and 12 months. **WOMAC score:** Significantly reduced after 6 and 12 months in both groups. There were no significant differences in WOMAC score between the intervention and control group but the intervention group tended to have a greater improvement than the control group. **SF-36:** Compared to baseline significantly improved after 6 and 12 months in the intervention group and only after 12 months in the control group. When comparing the intervention and control group the SF-36 score was significantly lower in the intervention group at both 6 and 12 months. **qMRI:** Compared to baseline the cartilage volume was significantly increased after 12 months only in the intervention group.
10	Lee et al [10]	RCT	12 / 12	II-IV	sIAI of AD-MSC	sIAI of 3 mL saline	VAS, WOMAC, KOOS, clinical examination (ROM, quadriceps	Incidence of AEs: 58% in the control group and 83% in the intervention group. Most common adverse events were mild to moderate arthralgia	**VAS, WOMAC, KOOS score:** Significantly improved after 6 months in the intervention group. **Clinical examination:** Compared to baseline the ROM was significantly improved after 6 months only in the intervention group.

							power, presence of joint effusion, presence of joint crepitus, presence of medial joint line tenderness, and presence of pes anserinus tenderness), X-ray, MRI (cartilage defect, modified Noyes grading system)	and joint effusion (resolved within 3 days). There were no clinically reports in the outcomes of physical examination, vital signs, and laboratory tests during the 6 months of follow-up. There were no SAE in either of the groups.	All other parameters were not significantly improved after 6 months in either group. **X-ray:** No changes after 6 months in either group. **MRI:** No significant change in cartilage defect in the intervention group after 6 months, but in the control group there were a significantly increase in the size of the cartilage defect after 6 months. When comparing the intervention group with the control group there were a significant difference in the amount of change in cartilage defect after the injection.
11	Freitag et al [11]	RCT	(1) group n=10, (2) group n=10 / 10	II,III	(1) group sIAI of AD-MSC, (2) group 2xIAI of AD-MSC, interval of 6 months	Conventional management only	VAS, WOMAC, KOOS, MRI (MOAKS)	Incidence of AEs: 60% in group (1) and 50% in group (2) with no significant differences in the incidence between groups. Most common adverse events were mild to moderate discomfort and/or swelling (self-limiting). Two participants reported pain and swelling for 4 weeks following injection. The second injection of MSC therapy at 6 months in group (2) was associated with a modest increase in reported moderate adverse events in comparison to the initial injection. No related SAEs were observed. NOTE A third treatment group receiving five injections of 40×10^6 AD-MSCs at baseline, 1, 2, 3 and 6 months, respectively, was intended but ceased due to observed and reproducible moderate adverse events (documented as increasing self-limiting pain with sequential injections at monthly intervals).	**VAS, WOMAC, KOOS score:** Significantly improved at all follow-up points in both group (1) and group (2). When comparing the two intervention groups and the control group the VAS, WOMAC and the KOOS scores were significantly improved in the intervention groups at 12 months. There were no significant differences between the intervention groups. **MRI (MOAKS):** 67% of participants within the control group had progression of cartilage loss with a further 56% having extension of osteophyte formation. By comparison, in group (1) only 30% of participants had further cartilage loss although 50% had progression of osteophyte formation at 12 months. In group (2) 89% of participants had improvement in cartilage or no progression in cartilage loss with stabilization of OA also indicated by 89% having no progression in osteophyte formation.
12	Yokota et al [12]	CS	(1) group n=42, (2) group n=38	II-IV	(1) group sIAI of AD-MSC or (2) group sIAI of 5 mL SVF	NA	VAS, KOOS, OMERACT-OARSI, ROM	Incidence of AEs: 2.3% in group (1) and 7.8% in group (2). Most common AEs were mild knee effusion/swelling (self limiting, except in one case in group (2) that was treated with 1-time knee aspiration). There were no SAEs in either of the groups.	**VAS, KOOS, OMERACT-OARSI score:** Compared to baseline, scores improved at all follow-up points in both groups but not significantly. There were no significant differences between the groups. **ROM:** Baseline and 6-month total ROM did not differ between the groups.

13	Zhao et al [13]	Double-blinded RCT	(1) low dose group n= 6, (2) mid dose group n= 6, (3) high dose group n= 6	II,III	2xIAI of AD-MSC for all dose groups, interval of 3 weeks	NA	WOMAC, SF-36, MRI (WORMS), qMRI	NS	**WOMAC, SF-36 score:** Compared to baseline, scores were significantly improved at 12 months in all groups. There were no significant differences between the groups. **MRI (WORMS) and qMRI:** Significant improvement was noted across all doses.
14	Soltani et al [14]	RCT	10 / 10	II-IV	sIAI of UC-MSC	sIAI of 10 mL saline	VAS, KOOS, ROM, MRA	Incidence of AEs: 40% in the intervention group and 0% in the control group. The AEs were local knee pain and mild effusion (resolved within 2-3 days). Re-examination at 2 weeks after treatment showed that the laboratory parameters all were unchanged. In the 6 months clinical and radiological follow-up, there was no ectopic mass formation or any other clinical adverse effects. There were no SAE in either of the groups.	**VAS score:** Not significantly improved at 6 months follow-up in either group. There were no significant differences between the groups. **KOOS score:** Compared to baseline, the KOOS score on multiple subscales significantly improved at multiple follow-up points in both groups. **ROM:** Compared to baseline, the ROM was improved at nearly all follow-up points except for the 2 weeks after the treatment visit in the intervention group. The ROM did not significantly improve at any follow-up point in the control group. **MRA:** Compared to baseline, the cartilage thickness increased significantly in the intervention group after 6 months. This increase was not observed in the control group.
15	Bastos et al [15]	RCT	(1) group n=16, (2) group n=14 / 17	I-IV	(1) group sIAI of BM-MSC, (2) group sIAI of BM-MSC + 10 mL auto PRP	sIAI of corticosteroid (4 mg of dexamethasone)	KOOS, ROM, Cytokine analysis of synovial fluid (IL-17A, IFN-gamma, human-TNF, human-IL10, human-IL6, human-IL4, and human-IL2)	NS	**KOOS score:** Compared to baseline, most KOOS subscale scores had significantly improved at 12 months follow-up in all groups. There were no significant differences between the groups but the intervention groups showed the highest improvements compared to the control group. **ROM:** Compared to baseline there were no significant improvements in ROM at any follow time point between the intervention and control group. **Cytokine analysis:** Compared to baseline a significant reduction in the levels of IL-10 was detected at 12 months follow-up, for all groups. Compared to baseline the control group also experienced a significant reduction in the levels of IL-17A at 12 months follow-up. There were no significant differences between the groups.
16	Matas et al [16]	RCT	(1) group n=10, (2) group n=10 / 9	I-III	(1) group 2xIAI of UC-MSC (interval of 6 months), (2) group 2xIAI of UC-MSC (baseline) and 5% AB plasma in 3 mL saline (6 months)	2xIAI of 3 mL HA (interval 6 months)	VAS, WOMAC, SF-36, OMERACT-OARSI, MRI (WORMS)	Incidence of AEs: 22% in the control group and 22% in both intervention groups (related to the first injection) for the most common AE (acute synovitis). In relation to the second injection the incidence of acute synovitis was 37.5% in the control group and 44% in both intervention groups. There were no significant differences between the AE	**VAS, WOMAC score:** Compared to baseline the VAS and WOMAC score were significantly improved after 12 months in the intervention groups. Compared to the control group (1) had statistically significant improvement in VAS and WOMAC after 12 months. Group (2) showed similar VAS and WOMAC scores as the control group towards final follow-up. **SF-36:** No changes in SF-36 score were detected in any group. **OMERACT-OARSI:** 100% in the intervention groups were found to be responders as opposed to 62.5% of patients in the control group, but this

								incidence across groups. Pain was the second most frequent AE without reaching statistical difference between groups. Both AEs were transient and were resolved by rest and oral acetaminophen. There were no SAE in either of the groups.	was not significant. **MRI:** Compared to baseline there was no change in WORMS score at any of the follow-up points in any of the groups. There were no differences between groups.
17	Bastos et al [17]	RT	(1) group n=9, (2) group n=9	1-4 (Dejour grade)	(1) group sIAI of BM-MSC, (2) group sIAI of BM-MSC+ 10 mL auto PRP	NA	KOOS	Incidence of AEs: 33% in group (1) and 22% in group (2). The adverse events were joint effusion, low back pain and mild-moderate knee pain (self-limiting without treatment or treated with dipyrone therapy). There was one SAE in both groups (intense knee pain treated with dipyrone therapy).	**KOOS score:** Compared to baseline, the KOOS score was significantly improved at 12 months follow-up in both groups. There were no significant differences between the groups.
18	Emadedin et al [18]	RCT	22 / 25	II-IV	sIAI of BM-MSC	sIAI of 5 mL saline suppl. with 2% HSA	VAS, WOMAC, Clinical examination (walking distance, painless walking distance, standing time with weight bearing, time to induction of stiffness (time to gelling), ROM)	Incidence of AEs: 100% in the treatment group and 24% in the control group. Most common AEs were mild-severe and related to musculoskeletal and connective tissue disorders. There was no SAE in either of the groups.	**WOMAC, clinical examination (painless walking):** Compared to baseline, the WOMAC total score, WOMAC pain and physical function subscales and painless walking distance were significantly improved at 6 months follow-up in both groups. When comparing the intervention and control group, the scores were significantly lowered in the intervention group at 6 months. **Other outcome measures:** Compared to baseline there were no significant changes in either group. There were no significant differences between groups.
19	Song et al [19]	Double-blinded, dose-ranging RCT	(1) low dose group n=6, (2) mid dose group n=6, (3) high dose group n=6	II,III	3xIAI for all dose groups, interval of 3 weeks and the last injection after 6 months	NA	NRS, WOMAC, SF-36, qMRI	Incidence of AEs: 66.67% group (1), 58.33% group (2) and 50% group (3). Most common AEs were transient mild to moderate knee pain and swelling (resolved within 7 days without special treatment). No changes on ECG, vital signs, physical examination and laboratory tests during the 12 months of follow-up. There were no SAEs in either of the groups.	**NRS score:** Compared to baseline, only significantly improved in group (3) throughout the entire follow-up period, and in group (1) after 3 months. **WOMAC:** Trend towards improvement throughout the whole follow-up period in all three groups. This improvement was significant in group (1) after 3 and 12 months, in group (2) after 3 months and in group (3) after 6 and 12 months. **SF-36:** Trend towards improvement throughout the whole follow-up period in all three groups. This improvement was significant in group (1) after 12 months and in group (2) after 18 months. **qMRI:** Compared to baseline there were a tendency to observe an increase in the cartilage volume throughout the whole follow-up period except after 24 months, in all groups.
20	Spasovski et al [20]	CS	9	IKDC system	sIAI of AD-MSC	NA	VAS, KSS, HSS-KS, T–L score, ROM, X-	A few days after injection, some patients reported moderate pain and swelling of the knee. These	**VAS, KSS, HSS-KS, T-L score:** Compared to baseline, these scores had significantly improved at 18 months follow-up for all patients. **ROM:** Not significantly changed

				B and D			ray, MRI (MOCART)	symptoms vanished within 1 week after treatment. No other side effects were noted during follow-up.	from baseline. **X-ray:** Compared to baseline no significant changes throughout the whole follow-up period. **MRI (MOCART):** Significantly improved at 18 months follow-up for all patients.
21	Kuah et al [21]	RCT	(1) low dose group n=8, (2) high dose group n=8 / 4	I-III	sIAI of AD-MSC for all dose groups	sIAI of 2 mL cell culture media and cryopreser-vative	VAS, WOMAC, AQoL-4D, Blood and urine samples for biomarker analysis [urine: type II collagen C2C peptide (C2C) and C-terminal telopeptide of type II collagen (CTX-II); serum: macrophage migration inhibitory factor (MIF), HA and C-terminal telopeptide of type I collagen (CTX-I), MRI (MOAKS), qMRI, activity level measurement	Incidence of the two most common AEs (arthralgia / joint effusion) were: 75% / 75% group (1), 100% / 37.5% group (2) and 100% / 75% in the control group. Vital sign monitoring, urinalyses, physical examinations, and ECGs were normal in the 12 months follow up period. 3 patients had abnormal blood work at follow-up (one in control group with slightly decreased neutrophils/white blood cell count, one in group (2) with slightly elevated liver enzymes, and one in group (2) with slightly elevated potassium). One patient had a severe AE (prepatellar bursitis) in group (1) (symptoms resolved with treatment in 2 weeks), another a moderate supra-patellar bursitis in group (2). There were no SAEs in either of the groups.	**VAS score:** Significantly improved in group (1) throughout the entire follow-up period and only after 3 months in group (2). **WOMAC pain, physical function and stiffness subscale scores:** Compared to baseline significant improvements were found in group (1) and (2) to a greater degree than in the control group at different follow-up time points. **AQoL-4D:** Compared to baseline, it was only group (1) which showed significant improvements at 12 months follow-up. **Biomarker analysis:** across the groups, there were few changes in the mean biomarker levels over the course of the trial, most within and between group results were not statistically significant. **MRI (MOAKS) scores:** Compared to baseline, MOAKS scores were similar between the groups at screening and the majority remained unchanged from screening to month 12 follow-up. **qMRI:** Compared to baseline, average lateral tibial cartilage volume decreased in group (2) and the control group whereas group (1) maintained its cartilage volume at 12 months follow-up. Other qMRI measurements (BMLs and cartilage defects) were similar between the groups with very few changes in these parameters over the course of the trial. **Activity levels:** Compared to baseline, the average activity levels remained consistent in each group, indicating no substantial change in activity levels during the trial.
22	Al-Najar el al [22]	Prospective open-label clinical trial	13	II,III	2xIAI of BM-MSC, interval of 4 weeks	NA	KOOS, qMRI	Incidence of AEs: 23%. Most common AEs were mild to moderate pain and swelling at the injection site (resolved within 48 hours). Physical examination and biochemical analysis were unchanged during follow-up. There were no SAE.	**KOOS score:** Significantly improved after 24 months follow-up. **qMRI:** Statistically significantly improved in the thickness of knee cartilage at 12 months follow-up.
23	Gupta et al [23]	RCT	(1) dose level I group n=10, (2) dose level II group n=10, (3) dose level III group n=10, (4) dose level	II,III	sIAI of BM-MSC for all dose group followed by sIAI injection of 2 ml HA (20 mg)	(5) 10 patients sIAI of 2 ml PLASMA-LYTE A, (6) 10 patients sIAI of 4 ml PLASMA-LYTE A, for all 20 patients this was followed by	VAS, WOMAC, ICOAP, x-ray, MRI (WORMS)	Incidence of AEs: 70% in group (1), (2), (3), (5) and 60% in group (4) and (6). Most common AEs were mild to moderate, being injection site pain, arthralgia knee pain and joint swelling (resolved with conservative / symptomatic treatment). Physical examination and vital signs data were unremarkable after the IAI.	**VAS score, WOMAC, ICOAP score:** Improved for all groups after 12 months, except for (4) group. The (1) group experienced the highest VAS, WOMAC and ICOAP scores improvement at 12 months compared to the control group and the (2) group. **X-ray:** No clinically meaningful changes in the X-ray parameters for any of the groups after 12 months follow-up. **MRI (WORMS):** Compared to baseline there were no perceptible change in WORMS score

			IV group n=10 / 20			sIAI injection of 2 ml HA (20 mg).		Hematology, serum chemistry, serology, urine analyses, and ECGs were normal in the 12 months follow up period. One patient in the (4) group had a SAE which was synovial effusion requiring hospitalization for one additional day of observation.	including cartilage signal and morphology for any of the groups at 12 months follow-up.
24	Pers et al [24]	Prospective, open-label, dose-escalating clinical trial	(1) low dose group n=6, (2) mid dose group n=6, (3) high dose group n=6	III-IV	sIAI of AD-MSC for all dose groups	NA	VAS, WOMAC, KOOS, PGA, SAS, SF-36, OMERACT-OARSI, qMRI, Histology of TKA or knee arthroscopy tissue after AD-MSC IAI in the follow-up period	Incidence of AEs: 100% group (1), 67% group (2) and 83% group (3) in the 0-3 months follow-up period and 100% group (1), 33% group (2) and 50% group (3) in the 3-6 months follow-up period. Most common AEs were slight knee pain/joint effusion (resolved with/without nonsteroidal anti-inflammatory drugs). No changes on ECG, vital signs, physical examination. A small increase in creatinine phosphokinase was observed in two patients and in alanine aminotransferase in one patient. There was also a mild decrease of neutrophil count in one patient who presented with a low baseline count and high variability of neutrophil count. OneSAE, unstable angina pectoris without increased cardiac markers, was reported in 1 patient at 3 months follow-up. There were no SAEs in either of the other groups.	**VAS, WOMAC, KOOS, PGA, SAS, SF-36, OMERACT-OARSI scores:** Compared to baseline, all groups experienced improvement in these outcome measures after 6 months follow-up. These improvements were only significant in group (1). **qMRI:** Compared to baseline, a possible cartilage improvement was observed in a limited amount of patients in all groups after 6 months follow-up. **Histology:** Histologic analysis of cartilage and synovium at 3 months showed signs of severe OA. Significant synovial inflammation was absent in two patients, whereas weak or moderate inflammation was observed in 9 patients. In one patient in group (1), a sheet of cells was observed. This finding could be interpreted as a stem cell graft on cartilage surface. None of the synovial or cartilage samples showed any tumor proliferation.
25	Soler et al [25]	Prospective open-label clinical trial	15	II,III	sIAI of BM-MSC	NA	VAS, WOMAC, HAQ, SF-36, Lequesne functional index, qMRI	Incidence of AEs: 100% of which the majority were mild-moderate. Most common adverse events were arthralgia and back pain from the extraction procedure (self-limited and lasted around 48–72 hours). No changes on ECG, vital signs, physical examination and laboratory tests during the 12 months of follow-up. There was one SAE (ovary cyst exertion), which was not related to the study medication.	**VAS, WOMAC, HAQ, Lequesne functional index scores:** Compared to baseline, outcome measurements were significantly improved after 12 months. **Long-term follow-up VAS score:** Compared to baseline, VAS score was further significantly improved after 48 months. **SF-36:** Only some of the subscale scores in the SF-36 were significantly improved after 12 months. **qMRI:** Compared to baseline a significant regeneration of cartilage was observed after 12 months.

26	Lamo-Espinosa et al [26, 27]	RCT	(1) low dose group n=10, (2) high dose group n=10 / 10	II-IV	sIAI of BM-MSC for both dose groups followed by sIAI injection of 4 ml HA (60 mg)	sIAI of 4 mL HA (60 mg)	VAS, WOMAC, ROM, X-ray, MRI (WORMS)	Incidence of AEs: 30% group (1), 60% group (2) and 10% in the control group. The AEs were articular pain requiring anti-inflammatory treatment during the first 24 hours. No changes on physical examination, vital signs and laboratory tests during follow-up period. There were no SAE in either of the groups.	**VAS, WOMAC score:** Significantly improved after 12 months only in the intervention groups. This improvement was still significant after 48 months compared to baseline but the effect had started to plateau compared to the 12 month follow-up. **ROM:** Compared to baseline, ROM was significantly improved already after 3 months in group (2) whereas this improvement was only seen after 12 months in group (1). The control group did not experience any ROM improvements. **X-ray:** Compared to baseline, the intervention groups and especially group (2) had no joint space reduction after 12 months follow-up whereas the joint space in the control group showed a borderline reduction. **MRI (WORMS):** Compared to baseline, group (2) showed a median improvement after 12 months follow-up. No sign of improvement was found in group (1) or the control group.
27	Vega et al [28]	RCT	15 / 15	II-IV	sIAI of BM-MSC	sIAI of 3 mL HA (60 mg)	VAS, WOMAC, Lequesne algofunctional indices, qMRI	Incidence of the most common AE (transient mild local pain and discomfort in the injected knee with inflammation and swelling during the first 1 to 7 days): 53% in the intervention group and 60% in the control group. This adverse event was resolved within a week with ibuprofen. There were no SAEs in either of the groups.	**VAS, WOMAC, Lequesne algofunctional indices scores:** Compared to baseline, these outcome measurements were significantly improved after 6 and 12 months in the intervention group, but not the control group. **qMRI:** Compared to baseline, a decrease in poor cartilage areas was observed in both groups after 12 months but this was only significant in the intervention group.
28	Jo et al [29, 30]	CS	(1) low dose group n=3, (2) mid dose group n=3, (3) high dose group n=12	III-IV	sIAI of AD-MSC	NA	VAS, WOMAC, KSS, KOOS, X-ray, qMRI, Histology on knee arthroscopy tissue after AD-MSC IAI in the follow-up period, Macroscopical changes to cartilage based on arthroscopy	Incidence of AEs during the first 6 months were: 67% group (1), 67% group (2) and 42% group (3). The most common AE was nasopharyngitis. No changes on physical examination, vital signs and laboratory tests during the first 6 months follow-up period. In group (1) there was one SAE: urinary stone, which the patient had a previous history of. After 2 years follow-up there were no clinically important adverse events according to physical examinations, vital signs, or laboratory tests.	**VAS score:** Significantly improved after 6, 12 and 24 months in group (3) and only at 24 months follow-up in group (1). **WOMAC score:** Significantly improved after 6, 12 and 24 months in group (3). **KSS score:** Significantly improved after 6, 12 and 24 months in group (1) and group (3). **KOOS score:** Compared to baseline, the KOOS score and the subscale scores were only significantly improved after 6, 12 and 24 months in group (3). In group (1), the subscale score KOOS-activity of daily living was significantly improved at 12 and 24 months. **NOTE:** A similar trend was observed in all the above-mentioned clinical measurements, showing an improvement from baseline but with a plateauing or decreasing effect after 1 year follow-up towards the 2nd year of follow-up. **X-ray:** Compared to baseline, there were no significant changes over the 2-year follow-up period in any of the groups. **qMRI:** Compared to baseline, the cartilage defect size and the size of the cartilage volume improved significantly in group (3) between 3 and 6 months. After 2 years, this improvement disappeared to a level similar to baseline.

									Compared to baseline, group (2) also significantly improved in cartilage volume after 6 months, but this improvement also disappeared after 2 years follow-up. For group (1), there were no significant improvements in the qMRI measurements during the 2 year follow-up period. **Arthroscopy:** Compared to baseline, regeneration of the lost cartilage was observed in the most severely degenerated areas in the knee in all groups at the 6 months follow-up arthroscopy. The regenerated cartilage looked glossy white with a smooth surface and with a probe it felt like healthy articular cartilage. Compared to baseline, a significant reduction in cartilage defect was only observed in group (3). This was also observed when using the ICRS cartilage injury classification. **Histology:** Biopsies taken from the regenerated articular cartilage areas showed integration with the subchondral bone. The cartilage demonstrated both collagen I and II fibrils. ICRS II (histology scoring system for the assessment of the quality of cartilage repair) changed significantly after AD-MSC injection on multiple parameters.
29	Orozco et al [31, 32]	CS	12	II-IV	sIAI of BM-MSC	NA	VAS, WOMAC, Lequesne indexes, SF-36, qMRI	Incidence of the most common AE during the 12 month follow-up (transient mild local pain and discomfort in the injected knee during the 1 to 6 days (controlled with ibuprofen) was 50%. There were no SAE after 24 months follow-up.	**VAS, WOMAC, Lequesne indexes scores:** Significantly improved after 3, 6, 12 and 24 months and did not change between 12 and 24 months. **SF-36:** Compared to baseline there were no significant changes after 12 months follow-up. No data were available from 24 months**. qMRI:** Compared to baseline a significant decrease of poor cartilage areas was observed after 24 months follow-up.
30	Emadedin et al [33]	Case study	6	IV	sIAI of BM-MSC	NA	VAS, WOMAC, Clinical examination (walking distance and time to induction of stiffness (time to gelling)), MRI	No local or systemic adverse events were reported during the one-year follow-up period.	**VAS, WOMAC, Clinical examination (walking distance and time to induction of stiffness (time to gelling)) score:** Compared to baseline, the VAS score, the joint functioning and the walking distance had improved during the follow-up period, but this improvement decreased towards 12 months follow-up. The other outcome parameters also tended to have the same improvement and decreasing patterns but not as pervasive. **MRI:** Compared to baseline, an increase in cartilage thickness, an extension of repair tissue over subchondral bone and a decrease in the size of edematous subchondral patches were observed in 3 out of 6 patients, after 6 months follow-up.
31	Davatchi et al [34, 35]	Case study	4 / 3	IV	sIAI of BM-MSC	NA	VAS, Clinical examination (Painless walking time, the number of stairs to climb, time	No local or systemic adverse events were reported during the follow-up period.	**VAS, Clinical examination (Painless walking time, the number of stairs to climb, time to induction of stiffness (time to gelling), ROM, patellar crepitus, swelling, knee instability, flexion contracture) score:** Compared to baseline all outcome measurements had improved after 6

							to induction of stiffness (time to gelling), ROM, patellar crepitus, swelling, knee instability, flexion contracture), X-ray		months but after this they gradually deteriorated. Outcome measures were however still better than baseline after 5 years follow-up. **X-ray:** No improvement of the joint space after 6 months follow-up.
32	Centeno et al [36]	Case series of injection in patients with various orthopedic diseases with complaint of pain in the knee, hip, foot-ankle, shoulder, lower back, hand/wrist and various other musculoskel-etal sites	Cohort 1 n=50 / Cohort 2 n=290	NA	Some patients received multiple IAI of BM-MSC	NA	Pain relief question, MRI (Cohort 1)	Incidence of the most common AEs (pain and swelling by injection site and other pain complaints) were 2% and 6%. Lab test: 3 patients reported transient abnormal bloodwork which all were self-limiting within a month. 1 patient had elevated calcium 3 months after BM-MSC injection, another had elevated TSH levels, and a third patient had increased liver function tests.<br>Some of the SAE's noted where: increasing severe knee pain after injection. The patient went through a thorough diagnostic work-up including arthroscopy however a conclusive cause was not established. After extensive physiotherapy the patient recovered. Tumor: 1 patient with a known history of elevated PSA and in an ongoing surveillance program for prostate cancer was diagnosed with prostate cancer 6 months following his BM-MSC injection. MRI 8 months post injection showed no tumor formation at the injection site and a cytogenetic analysis of his BM-MSCs showed normal karyotype.	**Pain relief question:** Compared to baseline, there was an average reduction of 46.9% after 11.3 months on the knee pain relief questionnaire in the knee OA group (n=133). **MRI:** Compared to baseline, no tumor formation at the BM-MSC injection sites were observed during any of the follow-up MRI examinations.
33	Centeno et al [37]	Case report	1	IV	IAI of BM-MSCs followed by 1 mL of auto nucleated cells suspended in PBS and 1 mL of 10% auto PL. Patients returned for 2	NA	VAS, FRI, ROM, qMRI	NS	**VAS, FRI, qMRI score:** Compared to baseline, the patient had significantly decreased VAS pain score, and increased ROM, as well as cartilage and meniscus growth on MRI at 6 months follow-up.

additional 10% intraarticu-lar knee PL injections (1mL) at week 1 and week 2 (post-transplant-ation). With the 2-week post-transplant PL supp., 1 mL of 10 ng/mL dexamethasone was also injected

AD = Arthroscopic debridement, AD-MSC = Adipose-derived mesenchymal stromal cells, AE = Adverse events, AQoL-4D = Assessment of Quality of Life 4D questionnaire, Auto = Autologous, BM-MSC = Bone marrow-derived mesenchymal stromal cells, CBC = Complete blood count, CS = Cohort study, ECG = Electrocardiogram, FRI = Functional Rating Index, HA = Hyaluronic acid, HAQ = Health Assessment Questionnaire, HSA = Human serum albumin, HSS-KS = Hospital for Special Surgery knee score, IAI = Intraarticular injection, ICOAP = Intermittent and Constant Osteoarthritis Pain, ICRS = International Cartilage Repair Society, IKDC = International Knee Documentation Committee subjective knee score, KL = Kellgren and Lawrence, KOOS = Knee injury and Osteoarthritis Outcome Score, KSS = Knee Society Score, MOCART = Magnetic Resonance Observation of Cartilage Repair Tissue, MOAKS = MRI Osteoarthritis Knee Score, MRA = Knee Magnetic Resonance Arthrography, MSC = Mesenchymal stromal cells, NA = Not applicable, NRS = Numerical Pain Rating Scale, NSAID = Non-Steroidal Anti-Inflammatory Drugs, NS = Not specified, OMERACT-OARSI = Outcome Measures in Rheumatology Osteoarthritis Research Society International, PBS = Phosphate Buffered Saline, PGA = Patient Global Assessment, PL = Platelet Lysate, PRP = Platelet Rich Plasma, PSA = Prostate specific antigen, qMRI = Quantitative MRI, RCT = Randomized Controlled Trial, ROM = Range of Motion, RT = Randomized Trial, SAE = Severe Adverse Events, SAS = Short Arthritis Assessment Scale, SF-36 = Short Form-36, sIAI = Single Intraarticular Injection, SVF = Stromal Vascular Fraction, Supplemented = Supp. T–L = Tegner–Lysholm Score, TKA = Total Knee Arthroplasty, TSH = Thyroid stimulating hormone, UC-MSC = Umbilical Cord-Derived Mesenchymal Stromal Cells, VAS = Visual Analog Scale, WOMAC = Western Ontario and McMaster Universities Osteoarthritis Index, WORMS = Whole-Organ Magnetic Resonance Imaging Score.

References

1. Kim, K.I., et al., Clinical Efficacy and Safety of the Intra-articular Injection of Autologous Adipose-Derived Mesenchymal Stem Cells for Knee Osteoarthritis: A Phase III, Randomized, Double-Blind, Placebo-Controlled Trial. Am J Sports Med, 2023. **51**(9): p. 2243-2253.
2. Günay, A.E., et al., Assessment of clinical, biochemical, and radiological outcomes following intra-articular injection of Wharton jelly-derived mesenchymal stromal cells in patients with knee osteoarthritis: A prospective clinical study. Medicine (Baltimore), 2022. **101**(37): p. e30628.
3. Samara, O., et al., Ultrasound-guided intra-articular injection of expanded umbilical cord mesenchymal stem cells in knee osteoarthritis: a safety/efficacy study with MRI data. Regen Med, 2022. **17**(5): p. 299-312.

4. Kim, K.I., et al., Safety and Efficacy of the Intra-articular Injection of Mesenchymal Stem Cells for the Treatment of Osteoarthritic Knee: A 5-Year Follow-up Study. Stem Cells Transl Med, 2022. **11**(6): p. 586-596.
5. Li, J., et al., Efficacy of autologous bone marrow mesenchymal stem cells in the treatment of knee osteoarthritis and their effects on the expression of serum TNF-α and IL-6. J Musculoskelet Neuronal Interact, 2020. **20**(1): p. 128-135.
6. Lu, L., et al., Intra-articular injections of allogeneic human adipose-derived mesenchymal progenitor cells in patients with symptomatic bilateral knee osteoarthritis: a Phase I pilot study. Regen Med, 2020. **15**(5): p. 1625-1636.
7. Dilogo, I.H., et al., Umbilical cord-derived mesenchymal stem cells for treating osteoarthritis of the knee: a single-arm, open-label study. Eur J Orthop Surg Traumatol, 2020. **30**(5): p. 799-807.
8. Chahal, J., et al., Bone Marrow Mesenchymal Stromal Cell Treatment in Patients with Osteoarthritis Results in Overall Improvement in Pain and Symptoms and Reduces Synovial Inflammation. Stem Cells Transl Med, 2019. **8**(8): p. 746-757.
9. Lu, L., et al., Treatment of knee osteoarthritis with intra-articular injection of autologous adipose-derived mesenchymal progenitor cells: a prospective, randomized, double-blind, active-controlled, phase IIb clinical trial. Stem Cell Res Ther, 2019. **10**(1): p. 143.
10. Lee, W.S., et al., Intra-Articular Injection of Autologous Adipose Tissue-Derived Mesenchymal Stem Cells for the Treatment of Knee Osteoarthritis: A Phase IIb, Randomized, Placebo-Controlled Clinical Trial. Stem Cells Transl Med, 2019. **8**(6): p. 504-511.
11. Freitag, J., et al., Adipose-derived mesenchymal stem cell therapy in the treatment of knee osteoarthritis: a randomized controlled trial. Regen Med, 2019. **14**(3): p. 213-230.
12. Yokota, N., et al., Comparative Clinical Outcomes After Intra-articular Injection With Adipose-Derived Cultured Stem Cells or Noncultured Stromal Vascular Fraction for the Treatment of Knee Osteoarthritis. Am J Sports Med, 2019. **47**(11): p. 2577-2583.
13. Zhao, X., et al., Multi-compositional MRI evaluation of repair cartilage in knee osteoarthritis with treatment of allogeneic human adipose-derived mesenchymal progenitor cells. Stem Cell Res Ther, 2019. **10**(1): p. 308.
14. Khalifeh Soltani, S., et al., Safety and efficacy of allogenic placental mesenchymal stem cells for treating knee osteoarthritis: a pilot study. Cytotherapy, 2019. **21**(1): p. 54-63.
15. Bastos, R., et al., Intra-articular injection of culture-expanded mesenchymal stem cells with or without addition of platelet-rich plasma is effective in decreasing pain and symptoms in knee osteoarthritis: a controlled, double-blind clinical trial. Knee Surg Sports Traumatol Arthrosc, 2020. **28**(6): p. 1989-1999.
16. Matas, J., et al., Umbilical Cord-Derived Mesenchymal Stromal Cells (MSCs) for Knee Osteoarthritis: Repeated MSC Dosing Is Superior to a Single MSC Dose and to Hyaluronic Acid in a Controlled Randomized Phase I/II Trial. Stem Cells Transl Med, 2019. **8**(3): p. 215-224.
17. Bastos, R., et al., Intra-articular injections of expanded mesenchymal stem cells with and without addition of platelet-rich plasma are safe and effective for knee osteoarthritis. Knee Surg Sports Traumatol Arthrosc, 2018. **26**(11): p. 3342-3350.
18. Emadedin, M., et al., Intra-articular implantation of autologous bone marrow-derived mesenchymal stromal cells to treat knee osteoarthritis: a randomized, triple-blind, placebo-controlled phase 1/2 clinical trial. Cytotherapy, 2018. **20**(10): p. 1238-1246.
19. Song, Y., et al., Human adipose-derived mesenchymal stem cells for osteoarthritis: a pilot study with long-term follow-up and repeated injections. Regen Med, 2018. **13**(3): p. 295-307.
20. Spasovski, D., et al., Intra-articular injection of autologous adipose-derived mesenchymal stem cells in the treatment of knee osteoarthritis. J Gene Med, 2018. **20**(1).
21. Kuah, D., et al., Safety, tolerability and efficacy of intra-articular Progenza in knee osteoarthritis: a randomized double-blind placebo-controlled single ascending dose study. J Transl Med, 2018. **16**(1): p. 49.
22. Al-Najar, M., et al., Intra-articular injection of expanded autologous bone marrow mesenchymal cells in moderate and severe knee osteoarthritis is safe: a phase I/II study. J Orthop Surg Res, 2017. **12**(1): p. 190.
23. Gupta, P.K., et al., Efficacy and safety of adult human bone marrow-derived, cultured, pooled, allogeneic mesenchymal stromal cells (Stempeucel®): preclinical and clinical trial in osteoarthritis of the knee joint. Arthritis Res Ther, 2016. **18**(1): p. 301.

24. Pers, Y.M., et al., Adipose Mesenchymal Stromal Cell-Based Therapy for Severe Osteoarthritis of the Knee: A Phase I Dose-Escalation Trial. Stem Cells Transl Med, 2016. **5**(7): p. 847-56.
25. Soler, R., et al., Final results of a phase I-II trial using ex vivo expanded autologous Mesenchymal Stromal Cells for the treatment of osteoarthritis of the knee confirming safety and suggesting cartilage regeneration. Knee, 2016. **23**(4): p. 647-54.
26. Lamo-Espinosa, J.M., et al., Intra-articular injection of two different doses of autologous bone marrow mesenchymal stem cells versus hyaluronic acid in the treatment of knee osteoarthritis: multicenter randomized controlled clinical trial (phase I/II). Journal of Translational Medicine, 2016. **14**(1): p. 246.
27. Lamo-Espinosa, J.M., et al., Intra-articular injection of two different doses of autologous bone marrow mesenchymal stem cells versus hyaluronic acid in the treatment of knee osteoarthritis: long-term follow up of a multicenter randomized controlled clinical trial (phase I/II). J Transl Med, 2018. **16**(1): p. 213.
28. Vega, A., et al., Treatment of Knee Osteoarthritis With Allogeneic Bone Marrow Mesenchymal Stem Cells: A Randomized Controlled Trial. Transplantation, 2015. **99**(8): p. 1681-90.
29. Jo, C.H., et al., Intra-articular injection of mesenchymal stem cells for the treatment of osteoarthritis of the knee: a proof-of-concept clinical trial. Stem Cells, 2014. **32**(5): p. 1254-66.
30. Jo, C.H., et al., Intra-articular Injection of Mesenchymal Stem Cells for the Treatment of Osteoarthritis of the Knee: A 2-Year Follow-up Study. Am J Sports Med, 2017. **45**(12): p. 2774-2783.
31. Orozco, L., et al., Treatment of knee osteoarthritis with autologous mesenchymal stem cells: a pilot study. Transplantation, 2013. **95**(12): p. 1535-41.
32. Orozco, L., et al., Treatment of knee osteoarthritis with autologous mesenchymal stem cells: two-year follow-up results. Transplantation, 2014. **97**(11): p. e66-8.
33. Emadedin, M., et al., Intra-articular injection of autologous mesenchymal stem cells in six patients with knee osteoarthritis. Arch Iran Med, 2012. **15**(7): p. 422-8.
34. Davatchi, F., et al., Mesenchymal stem cell therapy for knee osteoarthritis. Preliminary report of four patients. Int J Rheum Dis, 2011. **14**(2): p. 211-5.
35. Davatchi, F., et al., Mesenchymal stem cell therapy for knee osteoarthritis: 5 years follow-up of three patients. Int J Rheum Dis, 2016. **19**(3): p. 219-25.
36. Centeno, C.J., et al., Safety and complications reporting on the re-implantation of culture-expanded mesenchymal stem cells using autologous platelet lysate technique. Curr Stem Cell Res Ther, 2010. **5**(1): p. 81-93.
37. Centeno, C.J., et al., Increased knee cartilage volume in degenerative joint disease using percutaneously implanted, autologous mesenchymal stem cells. Pain Physician, 2008. **11**(3): p. 343-53.
